# Impact of Fat Intake on Blood Glucose Control and Cardiovascular Risk Factors in Children and Adolescents with Type 1 Diabetes

**DOI:** 10.3390/nu13082625

**Published:** 2021-07-29

**Authors:** Chiara Garonzi, Gun Forsander, Claudio Maffeis

**Affiliations:** 1 Department of Surgical Sciences, Dentistry, Pediatrics and Gynecology, Section of Pediatric Diabetes and Metabolic Disorders, University of Verona, 37126 Verona, Italy; garonzi.chiara@gmail.com; 2 Department of Pediatrics, Institute for Clinical Sciences, Sahlgrenska Academy, University of Gothenburg, S-405 30 Gothenburg, Sweden; gun.forsander@pediat.gu.se; 3 Department of Pediatrics, Region Västra Götaland, Sahlgrenska University Hospital, Queen Silvia Children’s Hospital, S-416 50 Gothenburg, Sweden

**Keywords:** fat intake, diet, nutrition, type 1 diabetes, glycemic control, cardiovascular diseases, inflammation, microbiota

## Abstract

Nutrition therapy is a cornerstone of type 1 diabetes (T1D) management. Glycemic control is affected by diet composition, which can contribute to the development of diabetes complications. However, the specific role of macronutrients is still debated, particularly fat intake. This review aims at assessing the relationship between fat intake and glycemic control, cardiovascular risk factors, inflammation, and microbiota, in children and adolescents with T1D. High fat meals are followed by delayed and prolonged hyperglycemia and higher glycated hemoglobin A1c levels have been frequently reported in individuals with T1D consuming high amounts of fat. High fat intake has also been associated with increased cardiovascular risk, which is higher in people with diabetes than in healthy subjects. Finally, high fat meals lead to postprandial pro-inflammatory responses through different mechanisms, including gut microbiota modifications. Different fatty acids were proposed to have a specific role in metabolic regulation, however, further investigation is still necessary. In conclusion, available evidence suggests that a high fat intake should be avoided by children and adolescents with T1D, who should be encouraged to adhere to a healthy and balanced diet, as suggested by ISPAD and ADA recommendations. This nutritional choice might be beneficial for reducing cardiovascular risk and inflammation.

## 1. Introduction

Type 1 diabetes (T1D) is one of the most common chronic diseases of childhood [1,2]. Glycemic control, nutrition therapy and physical activity are the three cornerstones of T1D management. The main goals of the therapy are the maintenance of blood glucose within a proper range, close to normoglycaemia, with as low frequency of hypoglycemic and hyperglycemic episodes as possible, and reduction of macro- and micro-vascular complications [3,4,5,6].

Even though clinical manifestations of cardiovascular diseases (CVDs) generally appear in adulthood, the vascular damage might start early in T1D and evidence of subclinical CVD can be detected in adolescence [7]. In addition, youth affected by prediabetes or diabetes have an increased risk of metabolic disorders in adulthood, such as hypertension, dyslipidemia, and metabolic syndrome, predisposing to CVD [8]. Therefore, the prevention and early detection of cardiovascular risk factors are mandatory in young with T1D, as assessed in the American Diabetes Association (ADA) and the International Society for Pediatric and Adolescent Diabetes (ISPAD) guidelines. Periodical screening and eventually proper treatment for hypertension, dyslipidemia, smoking and nephropathy are recommended [6,9].

The most important cardiovascular risk factor in T1D is glycemic control, also adjusting for potential confounders [10]. Glycemic control is affected by diet and, in particular, diet composition, which contributes to the development of complications in individuals with T1D [11]. Nevertheless, the specific role of the intake of different nutrients is still a matter of debate, in particular fat intake [12,13,14,15,16].

Therefore, the aim of this review is to assess the relationship between macronutrient intake, and in particular lipid intake, and glycemic control, CVD, inflammation and modifications of microbiota, in young people with T1D.

## 2. Nutrition Guidelines and Adherence in Children and Adolescents with T1D

Current dietary recommendations for people with diabetes reflect guidelines for healthy eating for the general population. The ADA and ISPAD guidelines for children and adolescents with diabetes underline the importance of an individualized assessment of nutrition therapy and the related best distribution of macronutrient, aiming at improving glycemic control and lower cardiovascular risk [4,6]. ISPAD recommendations give the following thresholds as a guide: carbohydrate intake should be 45–50% of total daily energy intake, fat intake no greater than 30–35% (saturated fat < 10%), and protein intake 15–20%. Energy intake should be appropriate for optimal growth in children and adolescents and keeping an ideal body weight. Diet should be assorted with healthy foods, such as fruits, vegetables, dairy, whole grains, legumes and lean meat. Thus, great emphasis is given to the quality of nutrients consumed. Healthy sources of carbohydrate foods, foods with high content of fibers, the replacement of saturated fat with polyunsaturated and monounsaturated fat and finally low-fat animal-derived and vegetable protein sources should be encouraged, according to current guidelines. Instead, restrictions in one macronutrient are discouraged, due to the risk of growth compromising and nutritional deficiencies [4].

Several studies from different countries demonstrated a low adherence in meeting nutrition recommendations among T1D children and adolescents, and particular concern emerged regarding high total fat and saturated fatty acid (SFA) intake [17,18,19,20,21,22,23,24,25,26]. Whether people with T1D were closer to guidelines than controls is debated, since contrasting results are described [13,18,21,23]. A lower adherence to recommendations was associated to poorer glycemic control, i.e., glycated hemoglobin A1c (HbA1c) levels, and therefore to the potential CVD risk and complications [11,22,23]. Of note, following a regular meal pattern was associated with better glycemic outcomes [18]. Moreover, a recent study showed how diet has changed in a 10-year period, showing that children and adolescents with T1D consume a higher amount of protein and fat and a lower amount of carbohydrate and fiber compared to 10 years ago [27]. Noteworthy, this study was conducted in Italy and therefore results may not be exported in other populations with different nutritional habits. Nevertheless, rapid changes and a deterioration of dietary habits, especially among youth, have been reported worldwide [28].

Nutritional variations seen in youth with T1D tend to follow the changes in the eating habits of the general population and, in particular, of their peers, who frequently do not meet the recommendations either [27,28]. Finally, it is worth mentioning that the food intake is frequently misreported, and especially under-reported, by children and adolescents with T1D and this should be considered when addressing the matter [29].

## 3. Fat intake and Glycemic Control

### 3.1. Food Intake and Postprandial Glycemic Control

**Figure 1:**
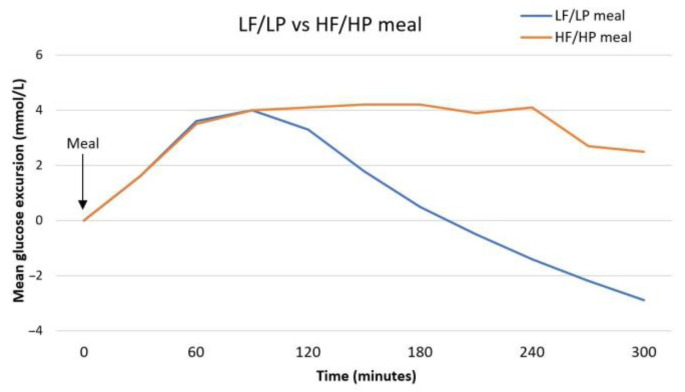
Mean glucose excursion after a low fat/low protein (LF/LP) or a high fat/high protein (HF/HP) meal. Reprinted with permission from [33]. Copyright 2013 by the American Diabetes Association [33].

Postprandial glycemic control is affected by food intake. It is mainly influenced by the amount of carbohydrate intake, along with insulin availability [30]. For this reason, guidelines recommend early nutrition education of individuals with T1D, including carbohydrate counting (CC), a meal planning approach based on the importance of carbohydrate in affecting postprandial glycaemia, used as a tool to improve glycemic control and facilitate flexible food choices [4,31,32]. Thus, insulin dosing at meals is generally decided upon the carbohydrate amount, often using insulin-to-carbohydrate ratio [31]. The importance of carbohydrate in affecting glycaemia has been known for long, however the impact of other diet macronutrients should also be considered. According to recommendations, to optimize postprandial glucose levels, other variables should be considered, including glycemic index, fat, protein and fiber intake [4]. It has been demonstrated that meals with high content of fat or protein lead to a delayed and prolonged increase in postprandial glycaemia, from 2 to 6 h after the meal, with small variations in ranges depending on the study considered [33,34,35,36]. An additive effect was reported when consuming high fat and high protein meals together [33]. Instead, early glycemic peak is reduced with high fat and high protein meals [37,38]. The differences in the mean glucose excursion after a low fat/low protein or a high fat/high protein meal are illustrated in Figure 1 [33]. Based on these findings, new methods to establish a more accurate need of insulin that would consider the complexity of the meal were required. Indeed, some studies showed a better glycemic control when using algorithms for calculating insulin dose that account also for protein and fat intake, besides carbohydrate [39,40]. However, more frequent episodes of hypoglycemia were reported when using supplementary fat/protein counting than CC [39]. To note, the Food Insulin Index (FII) is a new algorithm in which foods are sorted by the insulin response to an isoenergetic reference food in healthy people. Since food energy is used as the constant, all foods and their metabolic interactions could be included in the algorithm, allowing a broader assessment of insulin demand [41]. Its use has been compared to CC in adult studies, showing a better control in postprandial glycaemia in subjects with T1D using FII [42,43], also specifically for protein-containing food [41]. However, no significant changes in HbA1c levels and relatively high rates of mild hypoglycemia with both methods were described [41,43]. The efficacy of novel counting methods in children and adolescents with T1D need further studies to be established, since no clear benefit among one method to another was reported up to now [44]. Considering the variation of glycaemia after high fat and/or high protein meals, insulin dose adjustments are recommended [4]. Additional dose of insulin in dual wave bolus and/or the increase in percentage of insulin dose were studied [36,45,46,47,48], even if determining what strategy is more efficient in glycemic control must be further assessed. Thus, it is recommended to adapt meal insulin dose to counterbalance the delayed hyperglycemia resulting from high protein and high fat meals. To the best of our knowledge, available hybrid closed-loop insulin pumps do not have algorithms for fat and/or protein dosing. Considering the wide inter-individual differences in insulin dose demand for fat and protein, it is therefore important to individualize the treatment [4].

### 3.2. Fat Intake and HbA1c

As indicated in the latest ADA recommendations [6], HbA1c target levels must be individualized, but on a general level it has been shown that a HbA1c target of 6.5% (48 mmol/mol) gives a higher number of patients that achieve a good metabolic control with a higher percentage of glucose values in time in range (3.9–10 mmol/L) and time in target (4.0–8.0 mmol/L) without more episodes of hypoglycaemia [49].

Considering the role of fat intake on HbA1c, mixed results have been reported. Some studies did not find any association between total fat intake and glycemic control [50,51], while, on the contrary, other studies showed that the consumption of fat is associated with HbA1c levels. In particular, a cross-sectional study in 252 young people affected by T1D reported that there was a higher risk of having a suboptimal HbA1c between insulin pump users consuming the highest quartile of fat intake [12]. Another cross-sectional study among 114 children and adolescents with T1D showed that HbA1c levels were positively correlated with lipid intake and SFA and negatively correlated with monounsaturated fatty acid (MUFA) intake. Interestingly, when increasing the SFA intake of 1% of total energy, the risk of having HbA1c >7.5% increases by 53% [13]. A more recent study confirmed the results on MUFA intake, showing that a higher MUFA intake lowered the risk of having a HbA1c higher than 7.5%, independently from confounders [27]. The prospective Diabetes Control and Complication Trial showed an association between higher HbA1c concentrations and higher SFA, MUFA, and total fat intakes. Moreover, higher HbA1c concentrations were seen when substituting fat for carbohydrate intake, even though this association weakened after adjusting for baseline HbA1c and concurrent insulin dose [14]. In a behavioral nutrition intervention study in 136 adolescents with T1D, as regard lipids, a better glycemic control, i.e., lower HbA1c, was associated with lower percentage of energy from unsaturated fat intake, while no significant associations were found for total fat and SFA [52]. Another study reporting data from 1000 adults with T1D showed that MUFA intake was associated with higher variability in blood glucose measurements. When analyzing the macronutrient substitution, favoring fat intake over protein or favoring SFA over either MUFA or polyunsaturated fatty acids (PUFA) were associated with higher mean self-monitored blood glucose concentrations. However, these effects were no longer significant after adjusting for fiber intake. After that adjustment, it resulted that favoring either carbohydrate or fat over protein or favoring carbohydrate for fat were associated with higher glycemic excursions [53]. Finally, in adults with T1D, fat intake negatively correlated with time spent in euglycemia and positively correlated with time spent in hyperglycemia. To note, it was not correlated with time spent in hypoglycemia [15]. In this regard, the type of fat intake has shown different impact on hypoglycemic risk. In fact, while there was no correlation between daytime non-severe hypoglycemia and total or SFA intake, unsaturated fat was found to be protective of daytime hypoglycemia. Of note, when adjusting for total daily insulin dose per kilogram these associations were lost [54].

An adequate comparison between the abovementioned studies is difficult to obtain since they sometimes express results in different ways. However, it seems reasonable to assess that there is a relationship between lipid intake and glucose control in individuals with T1D. In particular, higher HbA1c levels have been more frequently reported by individuals having a high fat and SFA intake, while contrasting data are reported for unsaturated fatty acids. Further prospective studies are needed to clarify this issue.

### 3.3. Low-Carbohydrate (High-Fat) Diets

When addressing glycemic control, restrictive diets and particularly low-carbohydrate diets are worth mentioning, especially if considering the current arousing interest for such approaches. The rationale behind these diets is that several studies showed a worse glycemic control with higher carbohydrate intake in people with T1D and vice versa, i.e., better glycemic control with lower carbohydrate intake or lower glycemic-index diets [51,53,55,56,57]. However, in the general population it was demonstrated that both high and low percentages of carbohydrate diets were associated with increased mortality, with the lowest risk reported at 50–55% energy from carbohydrate [58].

There is no univocal definition of low-carbohydrate diets, since the term refers to different nutrition regimens that can be gathered as follows. Low-carbohydrate diets generally contain less than 100 g of carbohydrate per day, with macronutrient distribution amounting to 50–60% of fat, less than 30% of carbohydrate and 20–30% of protein. Very-low carbohydrate diets, with generally less than 50 g of carbohydrate per day, are ketogenic diets in which energy production depends on burning fat and the production of ketone bodies [59]. To guarantee energy requirements, a reduction of carbohydrate intake should be compensated by an increase of protein and lipid intakes. In detail, the individual needs to satisfy the energy requirement to maintain energy balance. The proportion of the different macronutrients may change but the total energy intake should guarantee total energy requirements. Therefore, if the amount of carbohydrate is reduced, an increase of fat and/or protein is necessary for compensating the energy reduction due to the lower carbohydrate intake. This may lead to a high-fat intake.

Recent reviews summarized the pros and cons of low-carbohydrate diets in people with T1D. Possible benefits of these diets are the improvement of glycemic control, the reduction of Hb1Ac levels and insulin requirement that may help to improve psychological outcomes, e.g., reducing diabetes distress and depressive symptoms [59,60,61,62]. In addition, low-carbohydrate diets may be a strategy for weight loss, if total energy intake is lower than the requirement [61]. Worth mentioning is a large online survey in youth and adults with self-reported T1D who followed a very-low carbohydrate diet (mean self-reported daily carbohydrate intake of 36 ± 15 g). The study reported HbA1c levels of 5.71% ± 0.58% in the pediatric age group, well below the ADA recommended target, and low rates of adverse diabetes-related medical events [63]. However, it is important to interpret those results with caution, mostly because of the self-selected sample and the self-reported data of carbohydrate intake and HbA1c levels [60,64]. Nevertheless, caution is needed with low-carbohydrate diets, and especially with very-low carbohydrate diets, because of the possible negative effects, such as the potential risk of diabetic ketoacidosis and oxidative stress, hypoglycemia and the reduced glucagon effect during hypoglycemia, the increase in saturated fatty acid consumption to maintain caloric intake and dyslipidemia, nutrient deficiencies and difficulties in maintaining these diets for long [59,60,61,62]. In growing children, low-carbohydrate diets may also negatively impact growth [59,61]. The unphysiological delivery of external insulin into the subcutaneous tissue instead of directly into the liver as in the non-diabetes situation leads in low-carbohydrate diets to more or less lack of insulin in the liver with e.g., less IGF-1 stimulation. Moreover, low-carbohydrate diets, as restrictive diets, may have adverse psychological outcomes, such as greater diabetes distress and augmented risk of eating disorders [60].

In summary, although low-carbohydrate diets and very-low carbohydrate diets may be effective in improving glycemic control, because of the potential important negative consequences of these diets, especially in children, we do not recommend the use of these diets for treating T1D, and caution is important when addressing to the topic [4,6,64].

## 4. Fat Intake and Cardiovascular Diseases

### 4.1. General Population

In the general population, the relationship between diet and CVD has been extensively explored. Focusing on lipid intake, it has raised harmful concerns for decades, since a high-fat dietary content, and in particular SFA, were correlated with elevated total and low-density lipoprotein (LDL) cholesterol, meaning a CVD risk [65]. In addition, it has been demonstrated that high total fat intake increases the risk of overweight and obesity [4]. However, increasing evidence has shown that there are no meaningful benefits in the restriction of total fat intake, particularly regarding the risk of coronary heart disease (CHD) and CVD mortality [66,67]. Thus, it seems to be more important to consider the quality of fat intake rather than the total amount. Trans fatty acids, which are mostly formed during the partial hydrogenation of vegetable oils, appear to confer an increased risk of CHD even at low levels of consumption. Considering the effects on serum lipids, they raise LDL and lower high-density lipoprotein (HDL) cholesterol concentrations, increasing the total cholesterol to HDL ratio, thereby contributing to the risk of CHD. They also increase triglycerides blood levels, in comparison with other fat intakes. The increased risk of CVD may be additionally explained by the fact that trans fatty acids have pro-inflammatory effects, and inflammation is an independent risk factor for atherosclerosis, sudden death from cardiac causes, diabetes and heart failure [68].

Since the amount of SFA consumption has been associated to the increased risk of CVD, current guidelines recommend limiting SFA intake [69,70]. However recent meta-analyses questioned the restrictions on SFA, since they have not found any association between the overall intake of total SFA and the risk of CHD or CVD [71,72]. Even though all SFA are usually described as a single group, studies are increasingly showing that different SFA and food sources of SFA have a different impact on cardiovascular risk. In fact, they can be gathered according to their length in short chain (containing 1–6 saturated carbons), medium chain (7–12 saturated carbons) and long chain fatty acids (13 or more carbons, either saturated carbons or containing one or more double bonds), leading to different absorption, transport and destination. No significant increase in the CHD risk was associated to the intake of short- to medium-chain saturated fatty acids (4:0–10:0), while it was associated to the intake of longer-chain saturated fatty acids (12:0–18:0) [73]. In addition, an inverse association between SFA intake and the risk of stroke was described, showing that a higher intake of SFA was associated to a lower risk of stroke [67].

When the SFA intake is lowered and replaced with unsaturated fat, in particular with PUFA, the incidence of CVD decreases [74,75,76]. Therefore, current guidelines conclude that there is strong evidence that replacing saturated with polyunsaturated fat in adults reduces the risk of CHD events and CVD mortality, while limited evidence is available about the substitution on SFA with MUFA and the improvement in CVD endpoints [70].

The intake of MUFA does not seem to be clearly associated with the risk of CHD [72], even if evidence is frequently ambiguous [77]. Data on a MUFA-rich diet has described an increase of HDL-cholesterol and a decrease in triglycerides, while no consistent data are available concerning the effect on total and LDL-cholesterol. The optimal amount of MUFA in diet is not yet established, and there is no consensus on recommendations [77].

PUFA can be divided into two groups, the *n*-6 PUFA (e.g., linoleic acid, arachidonic acid) and the *n*-3 PUFA (e.g., α-linoleic acid, eicosapentaenoic acid (EPA), docosahexaenoic acid (DHA)). PUFA intake may have a role in reducing CVD risk, by lowering the total to HDL cholesterol ratio, and a potential beneficial role in improving insulin resistance and reducing systemic inflammation [76].

In particular, *n*-3 PUFA consumption has shown several beneficial effects, including the decrease of plasma triglycerides, resting heart rate, and blood pressure, with possible improvements also in myocardial filling and efficiency, vascular function and decrease of inflammation. Strong evidence is reported concerning the reduced risk of cardiac death [78].

A recent Cochrane meta-analysis reported that a supplementation with long-chain *n*-3 PUFA lowers the risk of CHD deaths and events and reduces serum triglycerides levels. Instead, no significant reduction in CVD mortality, CVD events, or stroke were described. Moreover, the increase of α-linoleic acid is associated with a reduced risk of cardiovascular events and arrhythmia [79]. Otherwise, the effect of *n*-6 PUFA on CVD is at discussion. No clear evidence of reduction in CVD risk when increasing *n*-6 PUFA intake was seen in a Cochrane meta-analysis, so this question remains to be answered. Uncertainty remains also for the risk of stroke. However, the increased intake of *n*-6 PUFA was associated with a lower risk of myocardial infarction and a reduction in total cholesterol [80]. A high *n*-6 to *n*-3 PUFA ratio has raised concerns for a long time, in particular, on its inflammatory effects. However, no study seems to support the impact of higher levels of omega-6 than *n*-3 PUFA on inflammation or oxidative stress [81].

In conclusion, lipid intake affects CVD risk in different ways, depending on the fatty acid considered. The consumption of trans fatty acids has shown harmful effects, mostly on the risk of CHD. Although the overall SFA intake has been associated to the risk of CVD, increasing evidence is not confirming this association. However, evidence suggests a beneficial effect on CVD risk when the SFA intake is reduced and replaced with PUFA. In fact, PUFAs showed to lower the CVD risk, and in particular *n*-3 PUFAs, even if no clear evidence is available for *n*-6 PUFAs.

### 4.2. Individuals with T1D

Glycemic control plays a key role in preventing CVD in people with T1D, even though it is important to identify, and eventually manage, any additional risk factors [82]. The association between dietary intake and CVD risk has extensively been studied in the general population, but it may be different in individuals with T1D, because of the increased inflammation and the consequent CVD risk caused by the glucose variation and hyperglycemia [83]. Unfortunately, few studies reported this association in people with T1D, and in particular, children and adolescents. An 18-month intervention study on 136 youth with T1D described a positive association between SFA intake and HDL-cholesterol and between PUFA intake and the diastolic blood pressure (DBP). To note, authors suggest a role of omega-6 PUFA-derived eicosanoids in the stimulation of vasoconstriction, as a mechanism that might explain the association between PUFA intake and DBP. Thus, the authors of the study concluded that a lower intake of PUFA may be beneficial for CVD risk in youth with T1D [83]. Focusing on PUFA intake, a study investigated the consumption of PUFA from different sources and showed an inverse association between PUFA from nuts and LDL-cholesterol, while a positive one between PUFA from high-fat chicken and LDL. Therefore, when substituting nuts for chicken LDL cholesterol decreased [84]. Another study led on 180 children and adolescents with T1D assessed the impact of diet composition and cardiovascular risk [16]. It showed that non-HDL cholesterol, a predictor of CVD risk, was associated with lipids and lipid-to-carbohydrate intake ratio, while it was not associated with SFA, MUFA, PUFA and cholesterol intake. In addition, lipid-to-carbohydrate ratio gave an independent contribution to the inter-individual variability of non-HDL cholesterol at multiple regression analyses. Thus, the authors of the study suggested a stronger impact of total lipid intake than the different type of fatty acids on non-HDL cholesterol, even if a role of a higher saturated/unsaturated fat ratio cannot be excluded. An earlier study analyzed the connection between fat intake and serum lipid levels in people with T1D, showing that when consuming higher intakes of total fat, SFA and cholesterol, total and LDL-cholesterol levels increased, and thus the prevalence of CVD. Nevertheless, no relations were reported for HLD-cholesterol and triglycerides levels. Of note, those associations failed when adjusting for fiber intakes, which are independently associated with CVD. Moreover, higher fat intakes are frequently followed by low fiber intakes [85].

In conclusion, the relationship between lipid intake and CVD risk has been demonstrated also in individuals with T1D, although it might not completely correspond to that found in the general population. In particular, in people with T1D, higher amounts of total fat intake has been associated with an increased risk of CVD, while the relationship between different fatty acid intake seems to be less consistent and results are mixed. Further studies are needed to assess the role of different fatty acids on the cardiovascular risk in children and adolescents with T1D.

## 5. Fat Intake and Inflammation in Individuals with T1D

One of the mechanisms leading to vascular damage is inflammation [86]. Thus, we discuss the role of high-fat diets on inflammation and then specifically in people with T1D.

### 5.1. High-Fat Diets and Inflammation

The relationship between high-fat diets and inflammation has been recently reviewed, as well as the detailed mechanisms [87]. Diet composition is important for the activation of the immune system and thus to the systemic low-grade inflammation that is involved in the pathogenesis of several diseases, such as obesity, type 2 diabetes mellitus, atherosclerosis, non-alcoholic fatty liver disease and rheumatoid arthritis. In particular, the consumption of high-fat and/or high-carbohydrate diets cause postprandial inflammatory responses in healthy subjects, like the increase in plasma lipopolysaccharides (LPS), interleukin-6 (IL-6), tumor necrosis factor-α (TNF-α) levels and leukocyte counts, the induction of nuclear factor kappa-light-chain-enhancer-of activated B cells (Nf-κB) binding activity and reactive oxygen species generation. In addition, high-fat ingestion upregulated the expression of Toll-like receptors [87]. In vitro, SFA was shown to activate toll-like receptor 4 (TLR4) and elicit an inflammatory response. This effect was not demonstrated for unsaturated fatty acids that did not activate TLR4. This is probably because the antigenic part of LPS, the most important ligand of TLR4, is usually made up of SFA. The down-stream activation of Nf-κB can be abolished by *n*-3 PUFAs (DHA and EPA) [87,88]. The consumption of a high-fat meal also causes metabolic endotoxemia, in which LPS derived from gut microbiota were found in the circulation, resulting in a temporary pro-inflammatory state [87,89]. The acute postprandial inflammatory response can be considered physiological. Since nowadays people usually spend more than 16 h per day in the postprandial state, the resulting chronic low-grade inflammation may lead to the development of low-grade inflammatory diseases [87].

### 5.2. Inflammation in People with T1D

Inflammation is more pronounced in people with T1D, although available evidence is limited [90,91]. The SEARCH for Diabetes in Youth case control study [91] showed that IL-6 and fibrinogen were higher in youth with T1D than in controls, independently of glycaemia and obesity. High-sensitivity C-reactive protein (hsCRP), an acute-phase protein associated with systemic inflammation and an accurate marker of future atherosclerotic vascular disease [92], was significantly higher among youth with T1D with elevated levels of HbA1c (≥7.2%, 55.2 mmol/mol) and among normal-weight subjects [91]. Interesting is the association between elevated inflammatory markers and atherogenic lipid profile. In fact, in youth with T1D, higher hsCRP and fibrinogen correlated with higher total and LDL cholesterol and apolipoprotein B that may support the accelerated atherosclerosis process in T1D [91]. Another study consistent with the previous one, showed a low-grade inflammation, determined by elevated serum hsCRP levels, in children with T1D [93]. Soluble interleukin-2 receptor (IL-2R) and CD40 ligand were also found to be higher in subjects with T1D than in controls [90]. Importantly, inflammation is correlated to CVD and thus different inflammatory markers, like those mentioned above, were shown to predict CVD in individuals with T1D. As an example, low adiponectin levels, a protein secreted by adipose tissue, were correlated to both coronary artery disease and coronary artery calcification in those subjects. Other inflammatory markers associated with CVD in people with T1D are soluble interleukin-2 receptor, CRP, plasma fibrinogen, white blood cell levels, lipoprotein-associated phospholipase A2, serum endogenous secretory receptor for advance glycation end products, modified apolipoprotein B-rich immune complexes and connective tissue growth factor [90].

### 5.3. The Impact of Diet on Inflammatory Markers in People with T1D

Diet has shown to make an impact on inflammation in the general population, while few studies assessed this association in individuals with T1D. In the EURODIAB study, a lower consumption of fiber and PUFA and a higher consumption of cholesterol were associated with a greater low-grade inflammation, over the eight-year study period [94]. Another study showed that a diet closer to recommendations was associated to a lower level of low-grade inflammation in men, determined by lower hsCRP levels. Specifically, the study suggested that the consumption of fish, vegetables, fruits and berries, low-fat liquid milk products, and vegetable oil-based fats for spreads and for cooking, and limited intake of sweet pastry, candy, soft drinks, and salt may be beneficial in reducing systemic inflammation in T1D. The association between macronutrient intake and inflammation was not investigated in this study [95]. Bacterial endotoxins have a potential role in inflammation and the development of CVD. A study assessing the association between diet and serum LPS activity in individuals with T1D, showed that a healthy diet, made up of fish, fresh vegetables, fruits and berries might have positive effects reducing systemic endotoxemia. To note, no association between LPS activity and macronutrients was found, even for fat intake, contrasting with previous evidence in healthy subjects. The authors of the study speculated that this could be attributed to the fact that proportion of fat intake in other studies was higher than in this one, that other macronutrients may affect microbiota and endotoxemia and that the total energy intake may be a confounder in the previous studies results attributed to fat intake [96]. Adiponectin has anti-inflammatory and anti-atherogenic functions. Its levels were inversely associated with total SFA in a study performed in subjects with T1D, although further studies are needed to confirm this result [97]. In contrast, a recent study did not find the association between four different dietary quality indices considered and inflammation biomarkers in youth with T1D who participated in the SEARCH study for diabetes [98]. Overall, the association between diet and systemic low-grade inflammation shown in the general population needs to be further investigated in subjects with T1D. However, some evidence showed an inverse association between a PUFA intake and low-grade inflammation and between SFAs consumption and adiponectin levels (anti-inflammatory effects).

Thus, following a healthy diet might be beneficial also for lowering the inflammatory status, and therefore meeting the current dietary recommendations is relevant.

### 5.4. N-3 PUFA Supplementation in T1D Prevention and Treatment

Considering the autoimmune pathogenesis of the disease, some studies investigated the impact of omega-3 PUFA supplementation in the prevention and in the treatment of T1D. Available evidence has been recently reviewed. Both clinical and animal studies provided the hope that this intervention could be successfully used, even if further studies are needed to validate those findings. Several mechanisms are involved, such as the role of PUFAs’ metabolites in the modulation of the immune system. As an example, omega-6 PUFA-derived eicosanoids like arachidonic acid, synthetized by lipoxygenase, cyclooxygenase and cytochrome P450 enzymes, often show pro-inflammatory effects. On the contrary, EPA/DHA-derived eicosanoids and docosanoids show less inflammatory effects than omega-6-derived eicosanoids [99]. In contrast, those results were not confirmed in a meta-analysis that did not support that the PUFAs supplementation in children could modify the risk of clinical or preclinical T1D. Nevertheless, omega-3 PUFA intake in early life may lower the risk of preclinical T1D [100]. Overall, the reduction of omega-6 PUFAs and the increase of omega-3 PUFAs may be helpful in the prevention and treatment of T1D [99], even if more evidence is demanded.

## 6. Fat Intake and Microbiota

The human gut microbiota plays a key role in keeping local, systemic and brain homeostasis, particularly in the immunological, metabolic, structural and neurological fields [101,102]. It is mainly made up of about 10^14^ commensals bacterial, mostly belonging to the Firmicutes and Bacterioidetes (including *Bacterioides* and *Prevotella*) phyla [103,104]. Gut microbiota is highly influenced by environmental factors, including diet [105]. It largely takes nutrients from the fermentation of non-digestible dietary carbohydrate, resulting in the production of short-chain fatty acids (SCFAs), such as acetate, propionate and butyrate [106].

It has been suggested that gut microbiota of non-obese diabetic (NOD) mice plays a role in T1D pathogenesis. It was subsequently seen also in human studies, finding a strong association between gut dysbiosis and T1D pathogenesis [107]. Gut microbiota of both NOD mice and children affected by T1D has been showed to be limited or altered [105]. In particular, a case-control study conducted with 16 children with T1D and 16 controls, found marked differences in the fecal microbial composition, including a decreased number of Actinobacteria and Firmicutes with significantly increased Bacteroidetes, thus leading to a lower Firmicutes to Bacteroidetes ratio in children with T1D, compared to healthy controls. In particular, significantly increased levels of *Clostridium*, *Bacteroides* and *Veillonella* and a decrease in the number of *Lactobacillus*, *Bifidobacterium*, *Blautia coccoides*/*Eubacterium rectale* group and *Prevotella* were found in individuals with T1D [108]. Moreover, a significant reduction of *Faecalibacterium* was seen [105,109]. Thus, the significant reduction in butyrate producers, such as *Faecalibacterium*, and mucin degraders, such as *Prevotella*, along with a larger amount of other SCFA producers, such as *Bacterioides*, observed in the feces of those subjects, may contribute to the disease [105]. Butyrate, one of the SCFAs, is important for the epithelial integrity since it induces mucin synthesis and helps tight junction assembly that play a role in autoimmune diseases, like T1D. Instead, those effects are not seen with other SCFAs. Thus, it has been suggested that the ratio of butyrate-producing to other SCFAs-producing gut bacteria may be involved in T1D [110]. Moreover, the balance between the effectors of immune response (CD4^+^ T cells) and the regulatory ones (regulatory T cells (Treg)) is influenced by gut microbiota. In particular, commensal bacteria that stimulate regulatory responses and that are associated with an increased number of Treg, such as *Firmicutes*, *Lactobacilli*, and *Bifidobacteria*, are reduced in T1D. Otherwise, bacteria belonging to the phylum of Proteobacteria, including *Escherichia*, *Salmonella*, *Vibrio*, *Helicobacter*, *Yersinia*, *Legionellales* and other pathogens, frequently increased in the gut microbiota of people with T1D, stimulate the effectors of immune system. The consequent activation Th1 and Th17 may have very negative effects when persisting chronically [109]. Despite the described alterations of gut microbiota and its effects on T1D pathogenesis, further studies are demanded to assess the specific mechanisms involved in those interactions [107].

The effects of high-fatty acid diets in gut microbiota alterations have been reviewed recently. To note, little evidence is available up-to-now and most data comes from animal models. High-SFA diets have shown harmful effects on gut microbiota. High intake of SFA can increase the proportion of gut gram-negative bacteria, probably because of their higher tolerance of bile. In addition, they induce a dysbiosis characterized by reduced microbial diversity and increased Firmicutes/Bacteroidetes ratio. The increased abundance of Firmicutes promotes fatty acids absorption and lipid droplet formation. As mentioned before, high-fat diets also lead to endotoxemia, which may be facilitated by increased intestinal permeability. SFA diets showed higher LPS translocation through LPB (lipopolysaccharide-binding protein) and stronger inflammatory response, compared to unsaturated fatty acid rich diets in which higher LPS circulating levels have lower inflammatory potential probably because of the increased CD14 expression. In this regard, it is worth recalling that SFA can also directly stimulate TLR4. Thus, in addition to the concerning effects of high-SFA diets, also high-MUFA diets seem to have potential negative effects on gut microbiota. Otherwise, the potential negative effects of high-PUFA diets may be related to a higher intake of omega-6 compared to omega-3 PUFA [111].

Noticing the importance of gut dysbiosis, several studies tried to revert gut composition and reduce the risk of T1D. A study carried out in NOD mice showed that feeding them diets that promote the release of large amounts of the SCFA acetate and butyrate, through bacterial fermentation in the colon, provided a high protection from diabetes. In detail, acetate showed to decrease the frequency of autoreactive T cells and butyrate increased the number and function of Treg. Acetate- and butyrate-yielding diets improved gut integrity and lowered the concentration of diabetogenic cytokines in serum [112]. Other animal studies addressed the use of probiotic administration, showing promising results in T1D prevention [109]. Otherwise, few studies are available in humans. The prospective cohort study ‘TEDDY’ (The Environmental Determinants of Diabetes in the Young) showed that early probiotic supplementation (from 0 to 27 days of life) was associated with a decreased risk of developing T1D, in children with *DR3/4* genotype [113]. In addition, the administration of probiotics in adults affected by T1D showed a better glycemic control and the improvement of some factors associated to diabetic complications, such as metabolic syndrome, obesity, and lipid parameters [114]. In contrast, in another study the use of probiotics from birth to 6 months of life was not seen to prevent the development of T1D [115]. Thus, even if the role of gut microbiota in T1D has been shown in several studies, it is important to further assess possible interventions meant to ameliorate and reverse gut dysbiosis.

Overall, the alterations seen in people with T1D in gut microbiota should be taken into consideration when addressing this disease. In addition, the concerning effects of high-fat diets, and in particular the high intake of SFA, may also have an important impact in individuals with T1D, in which gut microbiota is already altered. Moreover, a healthy and balanced diet along with the potential benefit of probiotic consumption might be relevant measures in those subjects and may have a role in contrasting the gut microbiota alterations. However, further studies are demanded, as seen by the emerging roles of gut microbiota in many systems and diseases.

## 7. Conclusions

High-fat intake affects glucose control in children and adolescents with T1D, as suggested by the higher HbA1c levels frequently reported in individuals consuming high amounts of fat. Consistently, a delayed and prolonged increase of post-prandial glycaemia after high-fat meals, that requires adapting insulin dose to be counterbalanced, has also been demonstrated. Although a specific role of the different fatty acids on glucose control has been suggested, further evidence is necessary for providing recommendations.

A high-fat intake is associated with a pro-inflammatory post-prandial response through different mechanisms, including gut microbiota changes. These processes promote an increase of the cardiovascular risk that is already associated with diabetes.

Key relevant studies reported in this review are summarized in Table 1.

On the basis of this evidence, it is useful to promote a higher adherence to the dietary recommendations proposed by ISPAD and ADA to all children and adolescents with T1D [4,6] (Table 2), avoiding the intake of a high-fat diet.

## Figures and Tables

**Table 1:** Summary of the key relevant studies in individuals with T1D reported in this review.

Reference	Study Population (*n* *), Design	Key Findings
Smart et al., 2013 [33]	Pediatric (*n* = 33), four-by-four randomized crossover trial.	Meals with high content of fat or protein increase postprandial glucose excursion, 3–5 h after meal.
Balk et al., 2016 [50]	Adult ^1^ (*n* = 1659), 7-year prospective cohort analysis.	Total fat, SFA, PUFA and MUFA were not associated with HbA1c.
Lamichhane et al., 2015 [51]	Pediatric (*n* = 908), multicenter observational study.	No significant association between total fat and HbA1c.
Katz et al., 2014 [12]	Pediatric (*n* = 252), cross-sectional study.	Higher risk of suboptimal HbA1c between individuals consuming the highest quartile of fat intake.
Maffeis et al., 2012 [13]	Pediatric (*n* = 114), cross-sectional study.	HbA1c levels positively correlated with lipid intake and SFA and negatively correlated with MUFA intake.
Maffeis et al., 2020 [27]	Pediatric (*n* = 229), retrospective cross-sectional study.	Higher MUFA intake lowered the risk of having HbA1c higher than 7.5%.
Delahanty et al., 2009 [14]	Mixed ^2^ (*n* = 532), randomized, controlled clinical trial.	Higher fat, SFA and MUFA intake are associated with higher HbA1c levels.
Nansel et al., 2016 [52]	Pediatric (*n* = 136), behavioral nutrition intervention study.	Lower HbA1c was associated with lower unsaturated fat intake, no significant associations found for total fat and SFA.
Ahola et al., 2019 [53]	Adults (*n* = 1000), observational, cross-sectional study.	MUFA intake was associated with higher variability in blood glucose measurements.
Sanjeevi et al., 2018 [83]	Pediatric (*n* = 136), 18-months intervention study.	Positive association between SFA intake and HDL-cholesterol and between PUFA intake and diastolic blood pressure.
Maffeis et al., 2017 [16]	Pediatric (*n* = 180), cross-sectional study.	Non-HDL cholesterol was associated with lipids and lipid-to-carbohydrate intake ratio, not associated with SFA, MUFA, PUFA and cholesterol intake.
Snell-Bergeon et al., 2010 [91]	Pediatric ^3^ (*n* = 553 + 215 controls), cross-sectional, observational study.	Elevated inflammatory markers in youth with T1D, which were associated with an atherogenic lipid profile.
van Bussel et al., 2013 [94]	Adult (*n* = 491), prospective study.	Lower consumption of PUFA and higher of cholesterol were associated with a greater low-grade inflammation.
Ahola et al., 2017 [95]	Adult (*n* = 677), cross-sectional study.	A diet closer to recommendations was associated to a lower level of low-grade inflammation in men.
Liese et al., 2018 [98]	Pediatric ^4^ (*n* = 2520), observational, multicenter study.	No association between dietary quality indices and inflammation biomarkers.
Murri et al., 2013 [108]	Pediatric (*n* = 16 + 16 controls), case-control study.	Marked differences in the fecal microbial composition in individuals with T1D.

* Number of study subjects with T1D; ^1^ 15–60, ^2^ 13–39, ^3^ 10–22, ^4^ <20 years of age. HbA1c, glycated hemoglobin A1c; HDL, high-density lipoprotein; MUFA, monounsaturated fatty acid; PUFA, polyunsaturated fatty acids; SFA, saturated fatty acid; T1D, type 1 diabetes.

**Table 2:** Overview of the main ADA and ISPAD recommendations on nutrition management and macronutrients intake in children and adolescents with T1D.

Nutritional Recommendations on Diet Composition for Children and Adolescents with T1D
Individualized assessment of nutrition therapy and the related best distribution of macronutrient, aiming at improving glycemic control and lower cardiovascular risk [4,6].
As a guide: - carbohydrate intake: 45–50% of total daily energy intake, - fat intake no greater than 30–35% (saturated fat < 10%), - protein intake 15–20% [4].
Appropriate energy intake for optimal growth and keeping an ideal body weight [4].
Diet should be assorted with healthy foods, such as fruits, vegetables, dairy, whole grains, legumes and lean meat [4].
Restrictions in one macronutrient are discouraged (risk of growth compromising and nutritional deficiencies) [4].

ADA, American Diabetes Association; ISPAD, International Society for Pediatric and Adolescent Diabetes.

## Abbreviations

ADA	American Diabetes Association
CC	carbohydrate counting
CHD	coronary heart disease
CVD	cardiovascular diseases
DBP	diastolic blood pressure
DHA	docosahexaenoic acid
EPA	eicosapentaenoic acid
FII	Food Insulin Index
HbA1c	glycated hemoglobin A1c
HDL	high-density lipoprotein
HF/HP	high fat/high protein
hsCRP	high-sensitivity C-reactive protein
IL-6	interleukin-6
ISPAD	International Society for Pediatric and Adolescent Diabetes
LDL	low-density lipoprotein
LF/LP	low fat/low protein; LPS, lipopolysaccharides
MUFA	monounsaturated fatty acid
Nf-κB	nuclear Factor kappa-light-chain-enhancer-of activated B cells
NOD	non-obese diabetic
PUFA	polyunsaturated fatty acids
SCFA	short-chain fatty acids
SFA	saturated fatty acid
T1D	type 1 diabetes
TLR4	toll-like receptor 4
TNF-α	Tumor Necrosis Factor-α
Treg	regulatory T cells
